# Unexpected shunt-dependent hydrocephalus after unruptured aneurysm surgery—a case report

**DOI:** 10.1093/jscr/rjad415

**Published:** 2023-07-21

**Authors:** Myungsoo Kim, Byoung-Joon Kim, Sang-Youl Yoon, Youngseok Kwak

**Affiliations:** Department of Neurosurgery, School of Medicine, Kyungpook National University, Daegu, Republic of Korea; Department of Neurosurgery, School of Medicine, Kyungpook National University, Daegu, Republic of Korea; Department of Neurosurgery, School of Medicine, Kyungpook National University, Daegu, Republic of Korea; Department of Neurosurgery, School of Medicine, Kyungpook National University, Daegu, Republic of Korea

**Keywords:** Hydrocephalus, Unruptured aneurysm, Ventriculoperitoneal shunt

## Abstract

A chronic hydrocephalus after unruptured aneurysm surgery is an extremely rare condition. Its etiology and pathophysiology are also unclear. We report a case of chronic hydrocephalus in a patient who underwent permanent shunt placement after unruptured aneurysm clipping surgery. A 65-year-old man developed chronic hydrocephalus requiring shunt placement after clipping surgery of left anterior cerebral artery aneurysm and right middle cerebral artery aneurysm. This case shows that chronic hydrocephalus is a possible complication of unruptured aneurysm surgery, which can be resolved with an appropriate shunt operation.

## INTRODUCTION

A hydrocephalus that requires a permanent shunt placement after unruptured aneurysm surgery is an extremely rare complication, although it is a well-known sequela of ruptured aneurysm surgery. This complication occurs in 14–47% of aneurysmal subarachnoid hemorrhage [1–8].

To the best of our knowledge, this is the first case report of chronic hydrocephalus in a patient who underwent permanent shunt surgery after unruptured aneurysm clipping surgery. Perhaps because of its rare incidence, little attention has been paid to this condition. We report one case of shunt-dependent hydrocephalus among 1075 patients who underwent unruptured clipping surgery between January 2010 and April 2020 at a single institution.

## CASE REPORT

A 65-year-old right-handed man underwent magnetic resonance imaging (MRI) of the head to investigate vertigo, and two unruptured cerebral aneurysms were observed. The MRI showed no cerebral hemorrhage or hydrocephalus. The patient was neurologically intact. A cerebral angiogram showed a 2.7 mm left A1 segment aneurysm with a high aspect ratio, and a 1.7 mm right distal M1 segment aneurysm with an irregular wall.

We performed clipping surgery for both aneurysms through bilateral craniotomies. A bicoronal scalp incision and right supraorbital craniotomy were performed to expose the lesion to the right M1 segment. Proximal dissection of the Sylvian fissure revealed a small, red and fragile aneurysm arising from the right M1 segment of the middle cerebral artery. The right M1 aneurysm was clipped uneventfully. Subsequently, a left supraorbital craniotomy was performed. Dissection of the optico-carotid cistern and left Sylvian fissure revealed the aneurysm in the mid-portion of the A1 segment of the anterior cerebral artery. The aneurysm was small but had a high aspect ratio, which was also clipped uneventfully.

**Figure 1:**
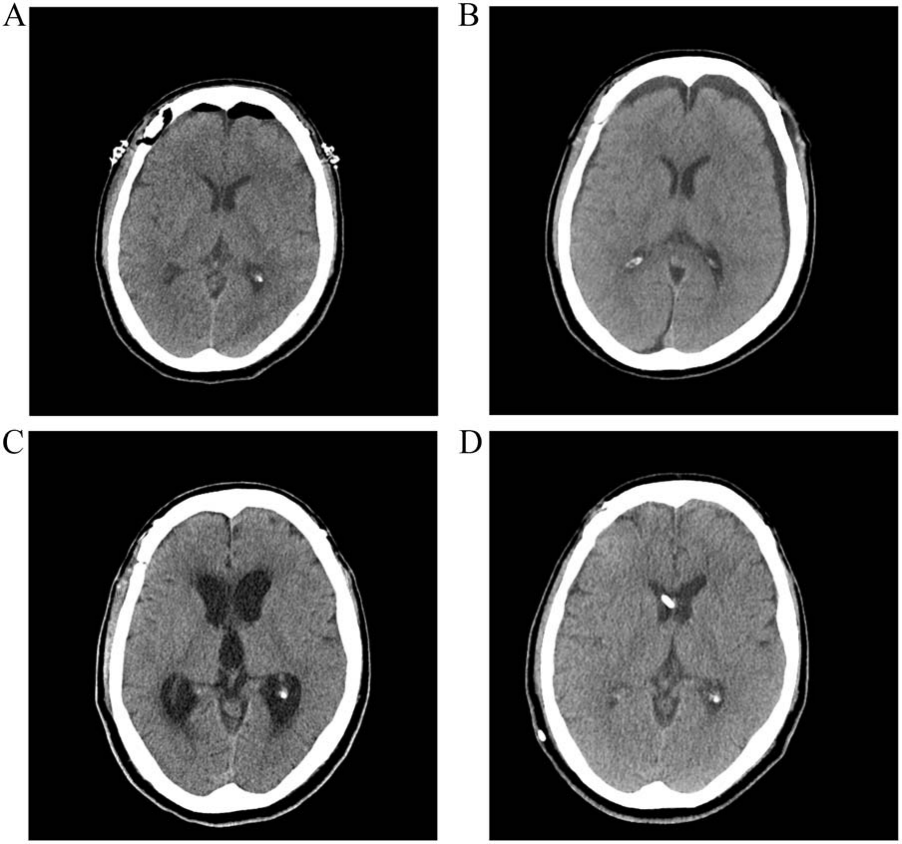
(**A**) Postoperative CT scan showing no subarachnoid or intraventricular hemorrhages. (**B**) Bilateral subdural fluid collection on postoperative day 13 CT scan. (**C**) Increased ventricle size by day 26. The patients developed symptoms of hydrocephalus. (**D**) A ventriculoperitoneal shunt was placed on day 47. Ventricle size decreased and clinical signs of hydrocephalus improved.

A postoperative computed tomography (CT) scan the day after surgery showed no postoperative hemorrhage (Fig. 1A). A postoperative CT angiogram showed successful clipping of both aneurysms. The patient was discharged on postoperative day 9. On postoperative day 13, the patient visited our clinic with a complaint of a severe headache. A CT scan showed subgaleal fluid collection on the left side, and scanty bilateral subdural fluid collection (Fig. 1B). We performed a lumbar tap of the cerebrospinal fluid (CSF), and the subarachnoid pressure was 12 cm H_2_O. The CSF was clear, and protein and glucose concentrations were 150.8 mg/dl and 52 mg/dl, respectively. The white blood cell count was 0 lymphocytes/mm^3^, and no microorganisms were identified in any culture. Blood tests showed no inflammatory reactions, such as leukocytosis or elevated C-reactive protein levels. On postoperative day 26, a follow-up CT scan showed improvement in the collection of the subgaleal and subdural fluids, and the headache subsided. However, the patient presented with gait disturbance and cognitive impairment 2 weeks later. A CT scan showed ventricular enlargement, and cisternography confirmed type IV hydrocephalus (Fig. 1C). The patient underwent permanent shunt placement and was discharged following good recovery (Fig. 1D).

## DISCUSSION

Data on unruptured aneurysms with shunt-dependent hydrocephalus are scarce because the incidence of chronic hydrocephalus in this group is very low. Few studies have investigated the incidence of shunting in unruptured aneurysms treated with endovascular coiling [9–14]. Edward M. *et al*. reported that the use of hydrogen coils on unruptured aneurysms has been associated with shunt-dependent hydrocephalus occurring in as many as 14% of the cases [10].

However, the incidence of shunting in unruptured aneurysms after clipping surgery has been poorly investigated. Hoh *et al*. alone investigated the incidence of shunt dependency in unruptured aneurysm surgery. They utilized a nationwide inpatient database and International Classification of Disease-9 code and performed cross-matching for diagnoses of unruptured and ruptured aneurysms with clipping and coiling. In their study, unruptured aneurysms had an incidence of 0.4% for shunt-dependent hydrocephalus in the clipping group, and 0.5% in the coiling group [15]. However, the attributed admission type for unruptured cases (elective or emergent) could have resulted from coding errors. In our study, the incidence of shunt-dependent hydrocephalus was 0.19% among the 1075 patients surveyed in a single institution for 10 years.

In ruptured aneurysm surgery, intraventricular hemorrhage is the major cause of chronic hydrocephalus requiring permanent shunt placement. Previous studies have shown that a large amount of subarachnoid blood seen on admission CT images is associated with the development of chronic hydrocephalus [16]. Although the precise mechanism of chronic hydrocephalus after a ruptured aneurysm is unclear, a generally accepted hypothesis is that it results from the impairment of CSF dynamics and CSF reabsorption through the arachnoid granulations [17, 18]. However, in our case, there was no rupture during the operation, and no subarachnoid or intraventricular hemorrhages were observed on the postoperative CT scan. Therefore, it is difficult to explain the mechanism of hydrocephalus in unruptured aneurysm surgeries.

Another possible cause of chronic hydrocephalus after surgery for an unruptured aneurysm is aseptic meningitis. Several cases of meningitis and related hydrocephalus after aneurysm coil embolization, use of hydrogel are reported [9, 11, 12, 19, 20]. Kouichi *et al*. reported elevated protein levels and pleocytosis in a CSF analysis. However, the glucose levels were normal and the cultures were negative, indicating non-specific aseptic meningitis and suggesting that long-term aseptic meningitis is involved in post-treatment hydrocephalus appearance [9]. In our patients, CSF protein levels were slightly elevated, but no significant pleocytosis and organisms were identified in any culture. These results provided no definite evidence of aseptic meningitis.

Although the mechanisms and pathophysiological effects of chronic hydrocephalus following unruptured aneurysm surgery are unclear, we hypothesize that the possible cause in our case was a broad tear of the arachnoid membrane and collection of the subdural fluid. Yoshimoto *et al*. suggested that decreased absorption of CSF and surgically created tears in the arachnoid membrane that communicate with the subdural space are involved in hydrocephalus development [21]. An arachnoid membrane tear could conceivably trap a significant amount of CSF by the flap-valve effect [21]. Furthermore, an arachnoid membrane tear can cause fibrosis and decrease CSF absorption, with consequent CSF accumulation and increased intracranial pressure, which may lead to postoperative hydrocephalus.

In this case series, two aneurysms at different locations were operated upon simultaneously, which caused tears of the Sylvian fissures and the broad arachnoid membrane. A tear in the arachnoid membrane gives rise to subdural fluid collection, which causes large volumes of CSF accumulation. Furthermore, arachnoid membrane tears can facilitate fibrosis and decrease CSF absorption.

## CONCLUSION

Chronic hydrocephalus in patients who undergo permanent shunt operation after unruptured aneurysm surgery is extremely rare. However, its etiology and pathophysiology remain unclear. Nevertheless, our case shows that chronic hydrocephalus is a possible complication of unruptured aneurysm surgery, which can be resolved with an appropriate shunt operation. For unruptured aneurysm surgery, clinicians should be aware that careful observation and treatment may be needed for potential chronic hydrocephalus.
